# Control of Wilt and Rot Pathogens of Tomato by Antagonistic Pink Pigmented Facultative Methylotrophic *Delftia lacustris* and *Bacillus* spp.

**DOI:** 10.3389/fpls.2016.01626

**Published:** 2016-11-07

**Authors:** Veeranan Janahiraman, Rangasamy Anandham, Soon W. Kwon, Subbiah Sundaram, Veeranan Karthik Pandi, Ramasamy Krishnamoorthy, Kiyoon Kim, Sandipan Samaddar, Tongmin Sa

**Affiliations:** ^1^Department of Agricultural Microbiology, Agricultural College and Research Institute, Tamil Nadu Agricultural UniversityMadurai, India; ^2^Korean Agricultural Culture Collection, National Academy of Agricultural Science, Rural Development AdministrationJeonju, South Korea; ^3^Department of Environmental and Biological Chemistry, Chungbuk National UniversityCheongju, South Korea; ^4^Department of Plant Pathology, Agricultural College and Research Institute, Tamil Nadu Agricultural UniversityCoimbatore, India

**Keywords:** methylotrophs, induced systemic resistance, pathogenesis-related proteins, biological control, tomato, antagonism

## Abstract

The studies on the biocontrol potential of pink pigmented facultative methylotrophic (PPFM) bacteria other than the genus *Methylobacterium* are scarce. In the present study, we report three facultative methylotrophic isolates; PPO-1, PPT-1, and PPB-1, respectively, identified as *Delftia lacustris, Bacillus subtilis*, and *Bacillus cereus* by 16S rRNA gene sequence analysis. Hemolytic activity was tested to investigate the potential pathogenicity of isolates to plants and humans, the results indicates that the isolates PPO-1, PPT-1, and PPB-1 are not pathogenic strains. Under *in vitro* conditions, *D. lacustris* PPO-1, *B*. *subtilis* PPT-1, and *B*. *cereus* PPB-1 showed direct antagonistic effect by inhibiting the mycelial growth of fungal pathogens; *Fusarium oxysporum* f. sp. *lycopersici* (2.15, 2.05, and 1.95 cm), *Sclerotium rolfsii* (2.14, 2.04, and 1.94 cm), *Pythium ultimum* (2.12, 2.02, and 1.92 cm), and *Rhizoctonia solani* (2.18, 2.08, and 1.98 cm) and also produced volatile inhibitory compounds. Under plant growth chamber condition methylotrophic bacterial isolates; *D*. *lacustris* PPO-1, *B*. *subtilis* PPT-1, and *B. cereus* PPB-1 significantly reduced the disease incidence of tomato. Under greenhouse condition, *D*. *lacustris* PPO-1, *B*. *subtilis* PPT-1, and *B*. *cereus* PPB-1 inoculated tomato plants, when challenged with *F*. *oxysporum* f. sp. *lycopersici, S*. *rolfsii, P*. *ultimum*, and *R*. *solani*, increased the pathogenesis related proteins (β-1,3-glucanase and chitinase) and defense enzymes (phenylalanine ammonia lyase, peroxidase, polyphenol oxidase, and catalase) on day 5 after inoculation. In the current study, we first report the facultative methylotrophy in pink pigmented *D. lacustris, B*. *subtilis*, and *B*. *cereus* and their antagonistic potential against fungal pathogens. Direct antagonistic and ISR effects of these isolates against fungal pathogens of tomato evidenced their possible use as a biocontrol agent.

## Introduction

Tomato (*Lycopersicon esculentum*) is one of the most popular commercial vegetable crops. In India, it occupies an area of 0.54 million ha with a production of 7.60 million tons (Kumar et al., 2012). Among the pathogens that affect the tomato crop, soil borne fungal pathogens, including *Fusarium, Pythium, Rhizoctonia*, and *Verticillium* causing the root rot or damping-off and wilt affect the quality with yield reduction (Lucas et al., 1997). Among these pathogens, *Fusarium oxysporum* f. sp. *lycopersici* is a highly destructive pathogen on both greenhouse and field grown tomatoes. In spite of the promising results shown by chemical treatments in controlling the fungal pathogens, phytotoxicity, and chemical residues are the major problems leading to environmental pollution and human health hazards. Alternatively in the present study, we have tested the possibility of using facultative methylotrophic bacteria which is ubiquitously occurring with intimate association with plants, as a biocontrol agent in controlling wilt and root rot pathogens of tomato.

Methylotrophs consists of a subpopulation of the bacteria which are able to use single carbon compounds (methanol and other C1 carbon compounds) as a sole carbon and energy source. Methylotropic bacteria can take up greenhouse gases and reduce global warming (Iguchi et al., 2015). Methylotrophs are found in both phyllosphere and rhizosphere of the plants and utilize the plant waste methanol as carbon source. Recently, there have been extensive studies on methylotrophs and their ability to promote plant growth were carried out (Madhaiyan et al., 2004; Poonguzhali et al., 2008; Yim et al., 2012). In addition, methylotrophic activity was reported in different bacterial families (Eyice and Schäfer, 2016). Among the facultative methylotrophic (FM) bacteria belonging to *Alpha*-, *Beta*-, and *Gamma*-subclasses of *Proteobacteria* and *Firmicutes*, the genus *Methylobacterium* has been widely studied. These FM bacteria ubiquitously occurring in plants can be isolated using selective media containing methanol as the sole carbon source and identified by their characteristic pink color (Corpe and Basile, 1982; Lidstrom and Chistoserdova, 2002). They are commonly referred as pink pigmented facultative methylotrophic (PPFM) bacteria. They are non-pathogenic and distributed widely in the plant phyllosphere, and have been isolated from more than 100 species of plants, ranging from liverworts and mosses to angiosperms and gymnosperms (Corpe and Basile, 1982).

Among the PPFM, the genus *Methylobacterium* is one of the dominant genera, which act as a symbiont with plants by consuming methanol, a plant metabolic waste and in turn providing vitamin B12, auxins, cytokinins useful for the plant growth (Madhaiyan et al., 2006). Although the genus *Methylobacterium* has also been well-documented for their induction of systemic resistance (ISR) in plants against plant pathogens (Madhaiyan et al., 2004, 2006; Indiragandhi et al., 2008), till date to the best of our knowledge there is no report available on the direct antagonistic effect of FM bacteria. Hence in the current study, we tested both direct antagonistic effect of FM bacteria against *Fusarium oxysporum* f. sp. *lycopersici, Sclerotium rolfsii, Pythium ultimum*, and *Rhizoctonia solani* causing tomato root rot and wilt diseases and indirect effect by the induction of ISR and pathogenesis related (PR) proteins in the pathogen challenged tomato plants grown under greenhouse condition.

## Materials and methods

### Source of bacterial strains, fungal pathogens, and tomato seeds

PPFM bacteria were isolated from the phylloplane of crop plants listed in Table 1 through leaf imprinting technique on solid ammonium mineral salts (AMS) medium (pH 6.8) supplemented with 0.5% methanol and cyclohexamide (30 μg ml^−1^) and incubated at 28°C for 3–5 days (Whittenbury et al., 1970). The single colonies with reddish pink pigmentation were picked and purified. The PPFM isolates were grown for 72 h on AMS medium. Gram reaction and other biochemical tests were carried out as described previously (Gerhardt et al., 1981). Plant pathogens (*Fusarium oxysporum* f. sp. *lycopersici* MTCC 4356, *S. rolfsii* KACC 43068, *R. solani* MTCC 4633, and *P. ultimum* KACC 40705) were obtained from Microbial Type Culture Collection, Chandigarh, India and Korean Agricultural Culture Collection, Jeonju, Republic of Korea. Fungal pathogens were grown and maintained in potato dextrose agar (PDA) before further use. Tomato cultivar CO_4_, which is susceptible to both seedling wilt and root rot, was obtained from the Department of vegetables at Horticultural College and Research Institute, Tamil Nadu Agricultural University, Coimbatore, India.

**Table 1:** *****In vitro*** antagonistic potential of pink pigmented facultative methylotrophic (PPFM) bacteria against plant pathogenic fungi and production of antimicrobial compounds**.

**Isolates**	**Source**	**Inhibition zone (cm)**				**Siderophore (μg ml^−1^)**	**Salicylic acid (μg ml^−1^)**	**β-1,3-glucanase (μg of glucose released min^−1^ mg of protein^−1^)**	**Chitinase (1 μmol of NAG released h^−1^ mg of protein^−1^)**
		***F. oxysporum* f. sp. *lycopersici***	***S*. *rolfsii***	***P*. *ultimum***	***R*. *solani***				
PPT-1	Tomato	2.05 ± 0.04^b^	2.04 ± 0.04^b^	2.02 ± 0.04^b^	2.08 ± 0.04^b^	27.45 ± 0.55^a^	88 ± 1.76^a^	278 ± 5.34^b^	4.95 ± 0.014^a^
PPN-1	Neem	1.85 ± 0.04^d^	1.64 ± 0.03^f^	1.12 ± 0.02^k^	1.38 ± 0.03^i^	20.54 ± 0.41^d^	71 ± 1.42^de^	118 ± 1.17^i^	3.89 ± 0.038^d^
PPB-1	Bhendi	1.95 ± 0.04^c^	1.94 ± 0.04^c^	1.92 ± 0.04^c^	1.98 ± 0.04^c^	25.44 ± 0.51^b^	87 ± 1.74^a^	265 ± 4.28^c^	4.80 ± 0.042^a^
PPBJ-1	Brinjal	1.75 ± 0.04^e^	1.14 ± 0.02^k^	1.42 ± 0.03^h^	1.68 ± 0.03^f^	23.19 ± 0.46^c^	77 ± 1.54^b^	178 ± 1.30^f^	3.39 ± 0.058^e^
PPG-1	Guava	1.25 ± 0.02^j^	1.54 ± 0.03^g^	1.82 ± 0.04^d^	1.48 ± 0.03^h^	18.45 ± 0.37^f^	61 ± 1.22^i^	140 ± 2.40^h^	3.86 ± 0.012^d^
PPL-1	Lime	1.65 ± 0.03^f^	1.24 ± 0.02^j^	1.52 ± 0.03^g^	1.18 ± 0.02^k^	20.02 ± 0.40^de^	70 ± 1.40^ef^	135 ± 2.18^h^	2.78 ± 0.038^g^
PPP-1	Prosophis	1.1 ± 0.02^k^	1.74 ± 0.03^e^	1.72 ± 0.03^e^	1.88 ± 0.04^d^	12.45 ± 0.25^i^	65 ± 1.30^h^	218 ± 4.54^e^	2.56 ± 0.052^h^
PPGR-1	Groundnut	1.55 ± 0.03^g^	1.44 ± 0.03^h^	1.22 ± 0.02^j^	1.58 ± 0.03^g^	17.32 ± 0.35^g^	73 ± 1.46^cd^	168 ± 3.15^g^	3.1 ± 0.023^f^
PPO-1	Onion	2.15 ± 0.04^a^	2.14 ± 0.04^a^	2.12 ± 0.04^a^	2.18 ± 0.04^a^	27.94 ± 0.56^a^	89 ± 1.78^a^	292 ± 3.65^a^	5.09 ± 0.111^a^
PPBO-1	Bougainvillea	1.45 ± 0.03^h^	1.84 ± 0.04^d^	1.32 ± 0.03^i^	1.78 ± 0.04^e^	17.45 ± 0.35^g^	68 ± 1.36^g^	186 ± 1.36^f^	3.50 ± 0.026^e^
PPPA-1	Parthenium	1.05 ± 0.02^l^	1.34 ± 0.03^i^	1.62 ± 0.03^f^	1.08 ± 0.02^l^	19.47 ± 0.39^e^	75 ± 1.50^bc^	234 ± 2.07^d^	4.2 ± 0.026^c^
PPGS-1	Grass	1.35 ± 0.03^i^	1.04 ± 0.02^l^	1.02 ± 0.02^l^	1.28 ± 0.03^j^	15.32 ± 0.31^h^	67 67 ± 1.34^gh^	224 ± 3.03^e^	4.5 ± 0.080^b^
LSD (*P ≤ 0.05*)		0.06	0.06	0.05	0.06	3.11	0.71	8.64	0.17

*None of the isolates produced HCN under tested condition; values are mean of three replications ± SD (standard deviation). Values in each column followed by same letter (s) are not statistically different by LSD (P ≤ 0.05)*.

### Antagonistic assay on solid medium

All PPFM bacterial isolates were screened for their ability to inhibit the growth of fungal pathogens on PDA plates by dual culturing technique with three replications (Yoshida et al., 2001). After 120 h incubation at 30 ± 2°C, the distance between the bacterial growth and fungal mycelial growth was measured to find out the antagonistic effect of PPFM bacterial isolates on test pathogenic fungi. The antagonistic effect of volatile antifungal compounds produced by PPFM bacterial isolates against test fungal pathogens was evaluated on PDA medium with three replications after 120 h of incubation at 30 ± 2°C as described by Trivedi et al. (2008). Briefly, PPFM isolates antagonism due to volatile compounds was evaluated by preparing a bacterial lawn on agar plates. After incubation for 24 h, the lid of the plate was replaced by a plate containing an agar block of the test fungus grown on PDA. The two plates were sealed together with parafilm. Control sets were prepared in a similar manner, without PPFM in the bottom plate. Then the Petri dishes were incubated at 28°C, and the observations were recorded after 5 days. Growth inhibition of the test fungus was calculated in % using the formula: (r1 − r2/r1) ^*^ 100, where r1 (a control value) represents the radial growth of the fungus in control sets without, and r2 with bacteria.

### Antagonistic assay in liquid medium

A volume of 1 ml of bacterial culture grown in potato dextrose broth for 72 h (10^9^ cfu ml^−1^) and a disc of test fungus (10 mm) from a well-grown fungal colony on PDA plates were inoculated into 50 ml potato dextrose broth in 250 ml conical flasks and incubated at 30 ± 2°C on a rotary shaker for 120 h. Potato dextrose broth inoculated only with fungi served as positive control. The broth with fungal mat in various treatments was passed through a pre-weighed Whatman No. 1 filter paper. The filter papers were dried for 24 h at 70 ± 1°C to obtain a constant weight. The percentage reduction in fungal mycelial weight was calculated as described previously by Trivedi et al. (2008).

### Other antimicrobial traits assay

Siderophore, hydrocyanic acid (HCN), and salicylic acid production, β-1,3-glucanase activity and chitinase activity of the isolates were measured as mentioned in Supporting Information File Materials and Method I.

### 16S rRNA gene sequence analysis

Bacterial isolates were grown in AMS medium for 72 h and DNA was extracted according to Sambrook et al. (1989). The gene encoding bacterial 16S rRNA was amplified through PCR with forward primer 27f: 5′-AGAGTTTGATCCTGGCTCAG-3′ and reverse primer 1492r: 5′-GGTTACCTTGTTACGACTT-3′. Nearly, complete 16S rRNA gene sequences of PPFM bacterial isolates from the automatic sequencer were aligned using the integrated SINA alignment tool from the ARB-SILVA website (Pruesse et al., 2007) and bacterial identities deduced by using the EzTaxon-e server (http://eztaxon-e.ezbiocloud.net/) to ascertain their closest relatives (Kim et al., 2012). Bacterial gyrA gene is a potential chromosomal marker for the phylogenetic identification of *Bacillus* genera. PCR amplification and detection of *gyr A* gene was given in the Supporting Information File Materials and Method II.

### Methanol utilization and detection of methanol dehydrogenase gene (mxaF)

Methanol utilization by the isolates was checked according to Sy et al. (2001) in M72 medium. The bacterial suspensions were diluted in M72 medium [optical density (OD) = 0.05], and added with one of the following compounds; methanol (MeOH) (10, 50, 100, or 500 mM), pyruvate (10 mM), or succinate (10 mM). Growth was monitored by measuring OD at 620 nm. Detection of mxaF gene was given in Supporting Information File Materials and Method III.

### Hemolytic activity and response to antibiotics

This experiment was conducted to test the production of exotoxins called hemolysin able to destroy the Red Blood Cells (RBC) and hemoglobin by the methylotrophic isolates *Delftia lacustris* PPO-1, *Bacillus subtilis* PPT-1, *Bacillus cereus* PPB-1 in sheep blood agar plates (catalog no. MP1301 Himedia, India). The plates were incubated for 48–72 h at 30°C and observed for presence of clear zone, greenish brown, and no zone which indicates the complete (β hemolysis), partial (α hemolysis), and no hemolytic (ɤ) activity, respectively. Susceptibility to the antibiotics rifampicin, ampicillin, oleandomycin, chloramphenicol, tetracycline, novobiocin, streptomycin, spectinomycin, kanamycin, trimethoprim, hygromycin, ceftriaxone, gentamycin, cefepime, and amikacin (each at 50 μg ml^−1^) was tested for *D*. *lacutris* PPO-1 on R2A agar plates as per Bauer et al. (1966).

### Plant growth chamber assay

Plant growth chamber experiment was conducted to assess the biocontrol potential of PPFM bacterial isolates on wilt and rot diseases of tomato. Bacterial cultures grown for 72 h were harvested by centrifugation at 10,000 × g for 10 min at 4°C, washed twice with sterile distilled water and suspended in 0.03 M MgSO_4_ solution. Tomato seeds were surface sterilized with 70% ethanol for 1 min, 0.5% NaOCl for 2 min, and washed four times with sterilized distilled water. The surface-sterilized seeds were imbibed in the 5 ml of bacterial suspension (10^9^ cfu ml^−1^) for 2 h, and single seed was sown in a 400 ml plastic pot filled with mixture of sterilized soil and farm yard manure in 3:1 ratio. The pots were subjected to following treatments; T1—*B*. *subtilis* PPT-1 + *F*. *oxysporum* f. sp. *lycopersici*, T2—*B*. *cereus* PPB-1 + *F*. *oxysporum* f. sp. *lycopersici*, T3—*D*. *lacustris* PPO-1 + *F*. *oxysporum* f. sp. *lycopersici*, T4—*B*. *subtilis* PPT-1 + *S. rolfsii*, T5—*B*. *cereus* PPB-1 + *S*. *rolfsii*, T6—*D*. *lacustris* PPO-1 + *S*. *rolfsii*, T7—*B*. *subtilis* PPT-1 + *R*. *solani*, T8—*B*. *cereus* PPB-1 + *R*. *solani*, T9—*D. lacustris* PPO-1 + *R*. *solani*, T10—*B*. *subtilis* PPT-1 + *P*. *ultimum*, T11—*B*. *cereus* PPB-1 + *P*. *ultimum*, T12—*D*. *lacustris* PPO-1 + *P*. *ultimum*, T13—*F*. *oxysporum* f. sp. *Lycopersici*, T14—*S*. *rolfsii*, T15—*R*. *solani*, T16—*P*. *ultimum* (Figure S1). One milliliter of fungal spore suspension (10^6^ spore ml^−1^) was applied to the soil on 30th day after sowing. Ten potted plants were maintained for each treatment. The experiment was conducted in a completely randomized block design with three replications. Pots were placed in growth chambers at 20 ± 1°C, photoperiod starting with 12 h dark followed by 12 h lights (18 μ mol m^−2^ s^−2^). Plants were watered with 1 X Hoagland solution. The disease incidence was observed by recording the wilting of leaves leading to plant drying. Randomly 15 plants were selected from each treatment and the number of wilted plants was recorded. The mean wilt disease incidence was expressed in percentage. Disease incidence was recorded up to 30 d after challenge inoculation with fungal pathogens. The percent disease incidence (PDI) was calculated, by using the formula. PDI = Number of plants infected/Total number of plants observed × 100.

### Greenhouse assay

Tomato seeds were surface sterilized and imbibed in the PPFM bacterial suspension as described earlier. Four seeds were sown in a 20 L earthen pot containing 10 Kg of unsterilized soil. Control seeds were imbibed with 0.03 M MgSO_4_ for 2 h. All treatments followed in the plant growth chamber assay were imposed. The experiment was conducted in a completely randomized block design with three replications. Twenty-one days after sowing, PPFM bacterial cells suspended in 0.03 M MgSO_4_ (10^9^ cfu ml^−1^) were sprayed over tomato plants with handheld pneumatic sprayer till the plants were completely wetted. Thirty days after sowing, 20 ml of fungal spore suspension (10^6^ spore ml^−1^) was applied into the soil. Initially, *F*. *oxysporum* f. sp. *lycopersici* (1.2 × 10^2^ cfu), *S*. *rolfsii* (1.5 × 10^2^ cfu), *R*. *solani* (2.1 × 10^2^ cfu), and *P*. *ultimum* (1 × 10^2^ cfu) were present in 1 g of experimental dry soil. Germination percentage was recorded on 7 days after sowing (DAS). Seedling vigor index was calculated as % germination × seedling length (shoot length + root length) in cm on 15 DAS (Baki and Anderson, 1973). Leaf samples were collected on 0, 3, 5, 7, and 9 days after challenge inoculation of pathogen and frozen immediately at −20°C for analyses of PR proteins and defense enzymes. The plant height was recorded on 30, 60, and 90 DAS. The crop was harvested at physiological maturity and yield was recorded.

### PR proteins analysis

Frozen leaves were ground at 4°C in an ice-chilled mortar with liquid nitrogen, and the resulting powder was suspended in 100 mM potassium phosphate buffer, pH 7.0 (2:2.5, w/v). Crude homogenates were centrifuged at 8000 × *g* for 10 min at 4°C, and the supernatants were kept frozen at −20°C until use. β-1,3-glucanase was assayed based on the release of reducing sugars from laminarin, as described by Liang et al. (1995). One unit of β-1,3-glucanase activity was determined as the amount of enzyme required to liberate 1 ng of glucose equivalent at 37°C in 1 h. Chitinase activity was assayed by measuring the release of *N*-acetyl-glucosamine from prepared colloidal chitin, as described by Singh et al. (1999). One unit of chitinase activity was determined as the amount of enzyme required to liberate 1 n mol of *N*-acetyl-glucosamine equivalent at 37°C in 1 h. PAL activity was measured using the procedure described by El-shora (2002). One unit represented the conversion of 1 μ mol of L-phenylalanine to cinnamic acid per min. Oxidative enzymes like PO and PPO were estimated as described previously (Compant et al., 2005). The activity of PO and PPO were expressed as the change in unit of absorbance at 420 nm/g of fresh weight per min. Catalase activity was assayed spectrophotometrically as described by Stern (1937). The activity was measured by monitoring the degradation of H_2_O_2_ using a spectrophotometer at 240 nm over 1 min. expressed in mmol min g^−1^ of leaf tissue^−1^.

### Statistical analysis

The data were analyzed by an analysis of variance (ANOVA) using the general linear model version 9.1; SAS Institute Inc., Cary, NC, USA. Means were compared using the least significant difference (LSD). The significance levels were within confidence limits of 0.05 or less.

## Results

### Antagonistic potential of PPFM bacterial isolates against fungal pathogens of tomato

Twenty PPFM bacteria were isolated from phylloplane of various crop plants (Table S1). Among the PPFM bacterial isolates, 12 isolates showed direct antagonism toward tested pathogens. The isolates PPO-1, PPT-1, and PPB-1 exhibited mycelial growth inhibition of *Fusarium oxysporum* f. sp. *lycopersici* (2.15, 2.05, and 1.95 cm), *S. rolfsii* (2.14, 2.04, and 1.94 cm), *P. ultimum* (2.12, 2.02, and 1.92 cm), *R. solani* (2.18, 2.08, and 1.98 cm), respectively, in a dual plate assay on day 5 (Table 1). All the tested methylotrophic isolates were able to produce siderophores and salicylic acid and none of the isolates produced hydrocyanic acid. The isolates PPO-1, PPT-1, and PPB-1 produced the maximum siderophore (27.94, 27.45, and 25.44 μg ml^−1^) and salicylic acid (89, 88, and 87 μg ml^−1^), respectively (Table 1). Chitinase and β-1,3-glucanase activities were observed higher in PPO-1 isolate followed by PPT-1 and PPB-1 isolates (Table 1).

**Figure 1:**
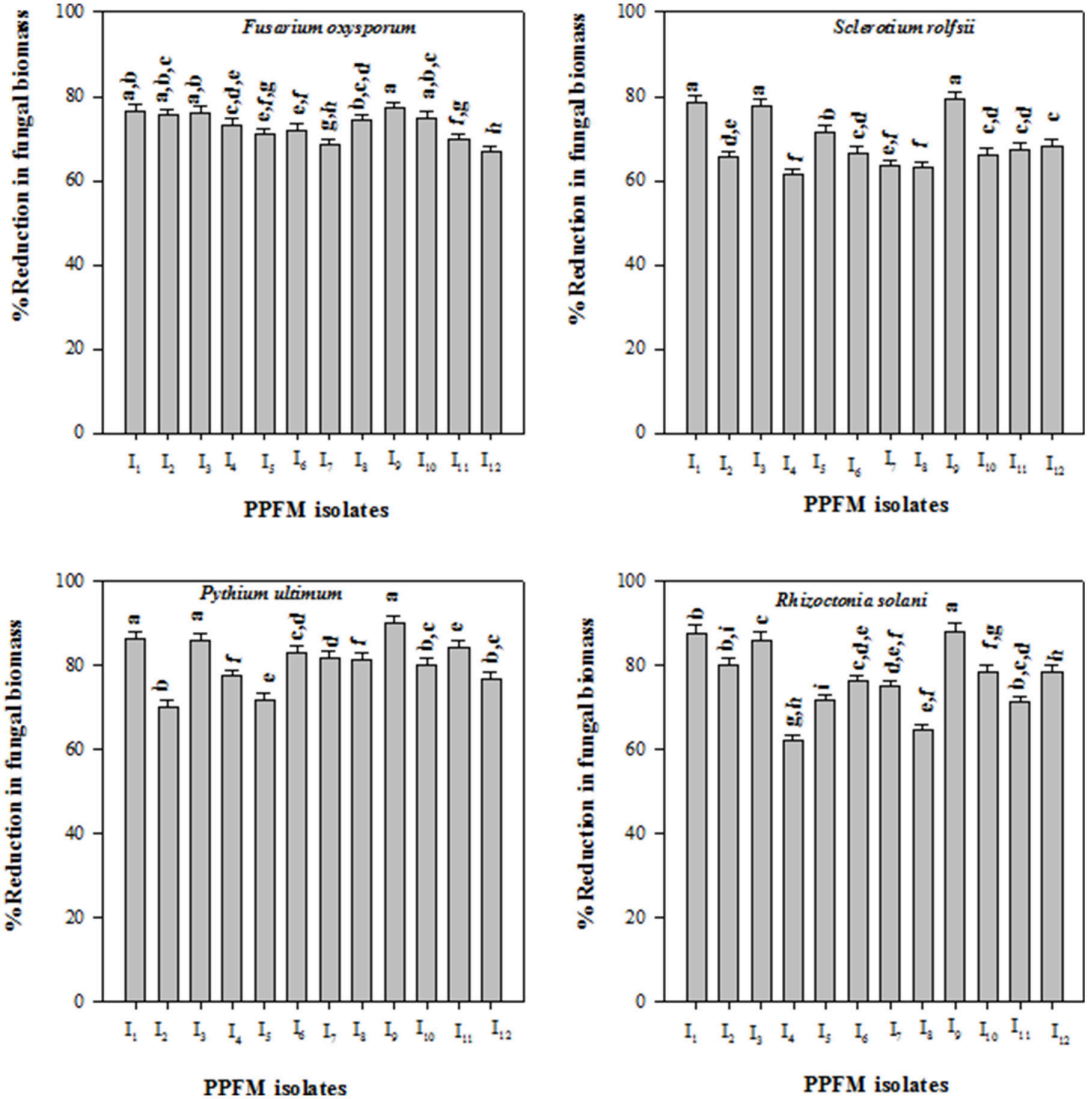
**Direct antagonistic effect of pink pigmented facultative methylotrophic isolates (I_**1**_–I_**12**_)**. I_1_, PPT-1; I_2_, PPN-1; I_3_, PPB-1; I_4_, PPBJ-1; I_5_, PPG-1; I_6_, PPL-1; I_7_, PPP-1; I_8_, PPGR-1; I_9_, PPO-1; I_10_, PPBO-1; I_11_, PPPA-1; I_12_, PPGS-1 against fungal pathogens in potato dextrose broth. Each value represents means of three replicates per treatment. Error bars indicate ± standard error (*SE*). In the bar, significant differences according to LSD at 0.05% levels are indicated by different letter(s).

All PPFM bacterial isolates produced volatile antifungal compound(s), and inhibited the mycelial growth of the tested fungal pathogens in sealed Petridishes. The higher mycelial growth inhibitions were observed in *F*. *oxysporum* f. sp. *lycopersici* (52.84, 51.73, and 50.65%), *S*. *rolfsii* (43.34, 42.23, and 41.12%), *P*. *ultimum* (57.78, 56.67, and 55.56%), and *R*. *solani* (44.45, 43.34, and 42.23%) due to the antagonistic effect of respective PPO-1, PPT-1, and PPB-1 isolates (Table 2). All PPFM bacterial isolates showed the reduction in pathogenic fungal biomass in the co-cultured liquid medium. The isolates PPO-1, PPT-1, and PPB-1 showed respective biomass reduction of *F*. *oxysporum* f. sp. *lycopersici* (77.14, 76.57, and 76.00%), *S*. *rolfsii* (79.26, 78.45, and 77.64%), *P*. *ultimum* (88.08, 87.65, and 85.95%), *R*. *solani* (89.81, 86.03, and 85.66%) (Figure 1).

**Table 2:** **Effect of facultative methylotrophic bacterial volatile antimicrobial compounds on the growth of fungal pathogens**.

**Inhibition (%)**				
**Isolates**	***F*. *oxysporum* f. sp. *Lycopersici***	***S*. *rolfsii***	***P*. *ultimum***	***R*. *solani***
PPT-1	51.73 ± 1.03^ab^	42.23 ± 0.84^ab^	56.67 ± 1.13^ab^	43.34 ± 0.87^ab^
PPN-1	41.73 ± 0.83^jk^	31.12 ± 0.62^k^	47.78 ± 0.96^ij^	34.45 ± 0.69^gh^
PPB-1	50.65 ± 1.01^bc^	41.12 ± 0.82^bc^	55.56 ± 1.11^bc^	42.23 ± 0.84^cb^
PPBJ-1	45.06 ± 0.90^gh^	35.56 ± 0.71^gh^	50.00 ± 1.00^gh^	31.12 ± 0.62^j^
PPG-1	49.50 ± 0.99^cd^	38.89 ± 0.78^de^	53.34 ± 1.07^de^	41.12 ± 0.82^cd^
PPL-1	42.84 ± 0.86^ij^	32.23 ± 0.64^jk^	52.23 ± 1.04^ef^	37.78 ± 0.76^e^
PPP-1	48.39 ± 0.97^de^	36.67 ± 0.73^fg^	45.56 ± 0.91^k^	32.23 ± 0.64^ij^
PPGR-1	43.95 ± 0.88^hi^	34.45 ± 0.69^hi^	51.12 ± 1.02^fg^	40.00 ± 0.80^d^
PPO-1	52.84 ± 1.06^a^	43.34 ± 0.87^a^	57.78 ± 1.16^a^	44.45 ± 0.89^a^
PPBO-1	47.28 ± 0.95^ef^	40.00 ± 0.80^cd^	48.89 ± 0.98^hi^	36.67 ± 0.73^ef^
PPPA-1	40.62 ± 0.81^k^	37.78 ± 0.76^ef^	54.45 ± 1.09^cd^	33.34 ± 0.67^hi^
PPGS-1	46.17 ± 0.92^fg^	33.34 ± 0.67^ij^	46.67 ± 0.93^jk^	35.56 ± 0.71^fg^
LSD (*P ≤ 0.05*)	1.58	1.26	1.75	1.27

*Values are mean of three replications ± SD (standard deviation). Values in each column followed by same letter(s) are not statistically different by LSD (P ≤ 0.05)*.

### Molecular characterization and phylogenetic analysis

PPFM bacterial isolates PPO-1 (JN 088183), PPT-1 (JN 088185), and PPB-1 (JN 088184) showed the highest 16S rRNA gene sequence similarities to type strains of *D. lacustris* DSM 21246^T^ (100%), *B. subtilis* KCTC 13429^T^ (99.87%), and *B. cereus* ATCC 14579^T^ (99.67%), respectively. The colony morphology of pink pigmented methylotrophic isolates PPO-1, PPT-1, and PPB-1 are shown in Figure S2. Bacterial growth was noted on either pyruvate or succinate as the sole carbon source. Also, growth was obtained at a methanol concentration up to 500 mM. Presence of methanol dehydrogenase gene (mxaF) was examined using *Methylobacterium extorquens* AM1 as reference strain. An amplification product of the expected size, 560 bp, for all the isolates similar to reference strain *M*. *extorquens* AM1 were noted which indicated the presence of mxaF. The isolate PPO-1 was Gram negative and isolates PPT-1 and PPB-1 were Gram positive. Isolates PPO-1, PPT-1, and PPB-1 did not exhibit hemolytic activity and isolate PPB-1 grew on mannitol egg yolk polymyxin agar MYP agar forming white precipitation which is the characteristic feature for *B. cereus*. Presence of housekeeping gene (*gyr A*) in two isolates of *Bacillus* sp. (PPT-1 and PPB-1) was examined, PPT-1 showed positive (Figure S3). Methylotrophic isolates PPO-1, PPT-1, and PPB-1 were susceptible to the antibiotics such as rifampicin, ampicillin, oleandomycin, chloramphenicol, tetracycline, novobiocin, streptomycin, spectinomycin, kanamycin, trimethoprim, hygromycin, ceftriaxone, gentamycin, cefepime, and amikacin at the concentration of 50 μg ml^−1^. For hemolytic activity the PPFM isolates did not produce any clear or greenish zone around the colonies in sheep blood agar medium, which may indicates that the isolates are non-pathogenic to human.

### Biocontrol effect of PPFM bacterial isolates on tomato wilt and rot diseases

**Figure 2:**
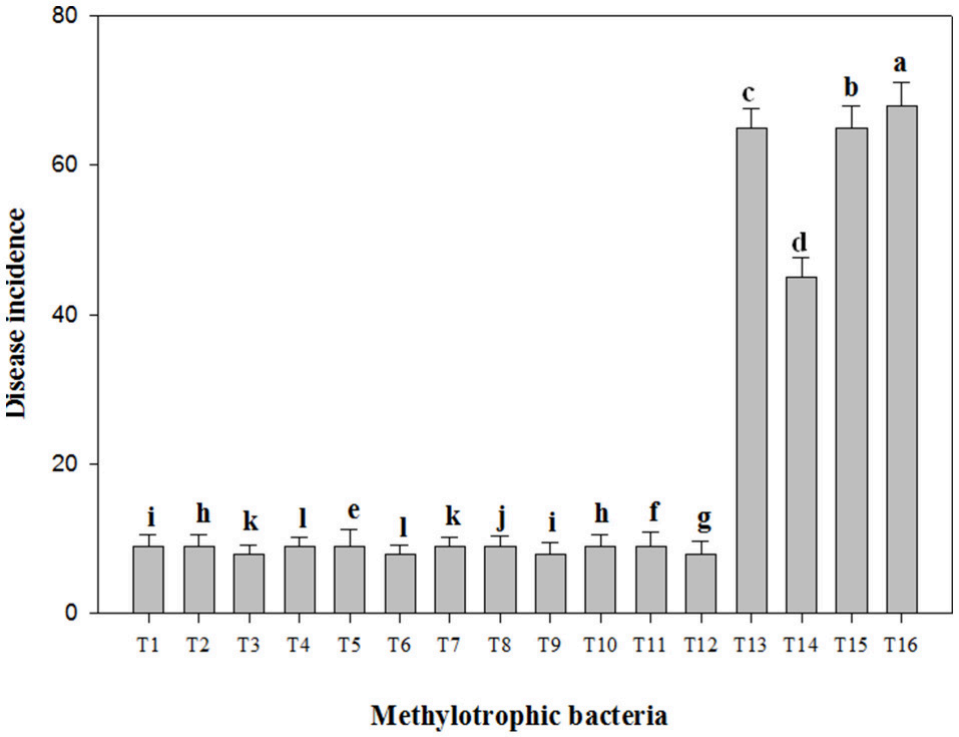
**Facultative methylotrophic bacterial inoculation effect on percent disease incidence in pathogen challenged tomato plants grown in growth chamber**. T1—PPT-1 + *F*. *oxysporum* f. sp. *lycopersici*, T2—PPB-1 + *F*. *oxysporum* f. sp. *lycopersici*, T3—PPO-1 + *F*. *oxysporum* f. sp. *lycopersici*, T4—PPT-1 + *S*. *rolfsii*, T5—PPB-1 + *S*. *rolfsii*, T6—PPO-1 + *S*. *rolfsii*, T7—PPT-1 + *R*. *solani*, T8—PPB-1 + *R*. *solani*, T9—PPO-1 + *R*. *solani*, T10—PPT-1 + *P*. *ultimum*, T11—PPB-1 + *P*. *ultimum*, T12—PPO-1 + *P*. *ultimum*, T13—*F*. *oxysporum* f. sp. *Lycopersici*, T14—*S*. *rolfsii*, T15—*R*. *solani*, T16—*P*. *ultimum*. Each value represents means of three replicates per treatment. Error bars indicate ± standard error (*SE*). In the bar, significant differences according to LSD at 0.05% levels are indicated by different letter(s).

Under plant growth chamber condition, soil inoculated with fungal pathogens showed 65% disease incidence. Higher diseases incidence was observed in the plants treated with *R*. *solani* followed by *P*. *ultimum, F*. *oxysporum* f. sp. *Lycopersici*, and *S*. *rolfsii*. However, the PPFM bacterial isolates inoculated tomato plants when challenged with the fungal pathogens recorded only 15% disease incidence (Figure 2). In greenhouse experiment, bacterization of tomato seeds with PPFM isolates *D*. *lacustris* PPO-1, *B. subtilis* PPT-1, and *B. cereus* PPB-1 significantly increased the germination percentage and the seedling vigor compared to control (Table 3). However, pathogen alone treatment has negative impact on seed germination, seedling vigor and plant height. No much variation was observed in plant height among the plants treated with different PPFM isolates and challenge inoculated with pathogens. However, significant variation in plant height was observed between PPFM challenge-inoculated with pathogen treatment and pathogen alone treated plants (Figure S4). *D*. *lacustris* PPO-1 inoculated tomato plants when challenged with *R*. *solani, S*. *rolfsi*, and *F*. *oxysporum* f. sp. *lycopersici* recorded the higher fruit yield of 9.35, 9.08, and 8.90 fruits plant^−1^, respectively (Table 3).

**Table 3:** **Effect of PPFM bacteria upon challenge inoculation of pathogens on growth and yield of tomato under pot culture condition**.

**Treatment**	**Germination percent**	**Vigor index**	**Plant height (cm)**			**No. of fruits**
			**30 DAS**	**60 DAS**	**90 DAS**	
T1—*B*. *subtilis* PPT-1 + *F*. oxysporum^*^	93^abcd^	1367.1^b^	25.90 ± 1.37^ef^	50.90 ± 2.59^d^	65.90 ± 3.49^e^	8.70 ± 0.17^def^
T2—*B*. *cereus* PPB-1 + *F*. oxysporum^*^	94^abc^	1343.2^b^	25.80 ± 1.37^ef^	50.80 ± 2.69^d^	65.80 ± 3.48^e^	8.50 ± 0.17^fg^
T3—*Delftia lacustris* PPO-1 + *F*. oxysporum^*^	94^abc^	1438.2^a^	27.30 ± 0.32^b^	52.30 ± 0.60^bcd^	67.30 ± 0.78^bcde^	8.90 ± 0.18^bcde^
T4—*B*. *subtilis* PPT-1 + *S*. *rolfsii*	92^bcd^	1365.2^b^	26.42 ± 1.40^cde^	51.92 ± 2.75^cd^	67.22 ± 3.56^cde^	8.87 ± 0.18^cde^
T5—*B*. *cereus* PPB-1 + *S*. *rolfsii*	91^cd^	1347.8^b^	26.32 ± 1.39^de^	51.82 ± 2.74^d^	67.12 ± 3.55^de^	8.67 ± 0.17^ef^
T6—*Delftia lacustris* PPO-1 + *S*. *rolfsii*	92^bcd^	1439.3^a^	27.85 ± 1.47^b^	53.35 ± 2.82^bc^	68.65 ± 3.63^bcd^	9.08 ± 0.18^bc^
T7—*B*. *subtilis* PPT-1+ *R*. *solani*	95^ab^	1361.3^b^	27.20 ± 1.44^bc^	53.45 ± 2.83^ab^	69.20 ± 3.66^ab^	9.14 ± 0.18^ab^
T8—*B*. *cereus* PPB-1 + *R*. *solani*	92^cd^	1356.6^b^	27.09 ± 1.43^bcd^	53.34 ± 2.82^bc^	69.09 ± 3.66^abc^	8.93 ± 0.18^bcd^
T9—*Delftia lacustris* PPO-1 + *R*. *solani*	96^a^	1445.3^a^	28.67 ± 1.52^a^	54.92 ± 2.91^a^	70.67 ± 3.74^a^	9.35 ± 0.19^a^
T10—*B*. *subtilis* PPT-1 + *P*. *ultimum*	91^cd^	1359.6^b^	24.09 ± 1.27^g^	47.34 ± 2.50^e^	61.29 ± 3.24^f^	8.09 ± 0.16^hi^
T11—*B*. *cereus* PPB-1 + *P*. *ultimum*	90^d^	1351.6^b^	23.99 ± 1.27^g^	47.24 ± 2.50^e^	61.19 ± 3.24^f^	7.91 ± 0.16^i^
T12—*Delftia lacustris* PPO-1 + *P*. *ultimum*	94^abc^	1442.6^a^	25.39 ± 1.34^f^	48.64 ± 2.57^e^	62.59 ± 3.31^f^	8.28 ± 0.17^gh^
T13—*F*. *oxysporum* f. sp. *lycopersici*	85^e^	1211.7^c^	19.75 ± 0.40^h^	28.75 ± 0.58^f^	34.75 ± 0.69^g^	4.30 ± 0.09^k^
T14—*S*. *rolfsii*	84^e^	1212.4^c^	18.25 ± 0.36^ij^	27.25 ± 0.55^fg^	33.25 ± 0.66^gh^	4.12 ± 0.08^kl^
T15—*R*. *solani*	83^e^	1210.2^c^	17.65 ± 0.35^jk^	26.65 ± 0.53^g^	32.65 ± 0.65^h^	4.20 ± 0.08^k^
T16—*P*. *ultimum*	84^e^	1213.5^c^	17.25 ± 0.34^k^	26.25 ± 0.52^g^	32.25 ± 0.64^h^	3.90 ± 0.08^l^
T17—Control	85^e^	1214.8^c^	18.50 ± 0.37^i^	27.5 ± 0.55^fg^	33.5 ± 0.67^gh^	7.51 ± 0.15^j^
LSD (*P ≤ 0.05*)	3.00	44.38	0.80	1.51	1.94	0.25

*Values are mean of three replications ± SD (standard deviation). Values in each column followed by same letter(s) are not statistically different by LSD (P ≤ 0.05)*.

* *F. oxysporum—F. oxysporum f. sp. lycopersici*.

In this study, *D*. *lacustris* PPO-1, *B*. *subtilis* PPT-1, and *B*. *cereus* PPB-1 inoculations induced significant protection in tomato plants against the tested pathogens. PPFM isolates *D*. *lacustris* PPO-1, *B*. *subtilis* PPT-1, and *B*. *cereus* PPB-1 inoculated tomato plants when challenge-inoculated with *F*. *oxysporum* f. sp. *lycopersici, S*. *rolfsii, P*. *ultimum*, and *R*. *solani*, increased PR proteins over uninoculated controls. The higher activities of β-1,3 glucanase, chitinase, PO, PPO, PAL, and catalase enzymes were recorded on 5th day after challenge inoculation with pathogens (Table 4). Increase in PO enzyme activity was observed in tomato plants inoculated with PPFM isolates *D*. *lacustris* PPO-1, *B*. *subtilis* PPT-1, and *B*. *cereus* PPB-1 and challenged with *F*. *oxysporum* f. sp. *lycopersici, S*. *rolfsii, P*. *ultimum*, and *R*. *solani*. The activities of PPO, PAL, chitinase, and catalase enzymes also had a similar trend (Table 4). In particular, *D*. *lacustris* PPO-1 inoculated tomato plants when challenge inoculated with *R*. *solani, P*. *ultimum, S*. *rolfsii*, and *F*. *oxysporum* f. sp. *lycopersici* exhibited significant increase in β-1,3-glucanase activity (Table 4). *Delftia lacustris* PPO-1 challenge inoculated with pathogen exhibited higher chitinase and catalase activity compared to other treatments.

**Table 4:** **Induction of defense enzymes in tomato against wilt and rot pathogens elicited by methylotrophic isolates under pot culture condition on day 5 after challenge inoculation of pathogens**.

**Treatment**	**Peroxidase activity (PO) (Changes in absorbance min^−1^ g of leaf tissues^−1^)**	**Polyphenol oxidase (PPO) (Changes in absorbance min^−1^g of leaf tissues^−1^)**	**Phenylalanine ammonia lyase (PAL) (μ mol of trans-cinnamic acid min ^−1^ g of leaf tissues^−1^)**	**β-1,3 glucanase activity (ng of glucose min ^−1^g of leaf tissues^−1^)**	**Chitinase activity (n mol of Glc NAc min ^−1^ g of leaf tissues^−1^)**	**Catalase activity (μ mol min^−1^g of leaf tissues^−1^)**
T1—*B*. *subtilis* PPT-1+ *F*. oxysporum^*^	1.23^bc^	0.94^efg^	0.84^d^	60.37^a^	124.77^b^	1.06^b^
T2—*B*. *cereus* PPB-1 + *F*. oxysporum^*^	1.22^c^	0.91^h^	0.84^d^	56.45^b^	122.08^b^	0.91^c^
T3—*Delftia lacustris* PPO-1 + F. oxysporum^*^	1.33^a^	0.97^bcd^	0.86^bcd^	58.77^a^	129.49^a^	1.16^a^
T4—*B*. *subtilis* PPT-1+ *S*. *rolfsii*	1.24^bc^	0.95^def^	0.85^cd^	60.3^9a^	124.79^b^	1.07^b^
T5—*B*. *cereus* PPB-1 + *S*. *rolfsii*	1.23^bc^	0.92^gh^	0.85^cd^	56.47^b^	122.10^b^	0.92^c^
T6—*Delftia lacustris* PPO-1 + *S*. *rolfsii*	1.34^a^	0.98^abc^	0.87^abc^	58.79^a^	129.51^a^	1.17^a^
T7—*B*. *subtilis* PPT-1 + *R*. *solani*	1.26^b^	0.97^bcd^	0.87^abc^	60.40^a^	124.80^b^	1.04^b^
T8—*B*. *cereus* PPB-1 + *R*. *solani*	1.25^bc^	0.94^efg^	0.87^abc^	56.50^b^	122.13^b^	0.89^c^
T9—*Delftia lacustris* PPO-1 + *R*. *solani*	1.36^a^	1.00^a^	0.89^a^	58.80^a^	129.54^a^	1.14^a^
T10—*B*. *subtilis* PPT-1+ *P*. *ultimum*	1.25^bc^	0.96^cde^	0.86^bcd^	60.41^a^	124.81^b^	1.05^b^
T11—*B*. *cereus* PPB-1 + *P*. *ultimum*	1.24^bc^	0.93^fgh^	0.86^bcd^	56.49^b^	122.12^b^	0.90^c^
T12—*Delftia lacustris* PPO-1 + *P*. *ultimum*	1.35^a^	0.99^ab^	0.88^ab^	58.81^a^	129.53^a^	1.15^a^
T13—*F*. *oxysporum* f. sp. *lycopersici*	0.76^d^	0.57^j^	0.68^f^	39.67^c^	102.40^c^	0.61^d^
T14—*S*. *rolfsii*	0.77^d^	0.58^ij^	0.69^ef^	36.69^d^	102.42^c^	0.62^d^
T15—*R*. *solani*	0.79^d^	0.60^i^	0.71^e^	39.70^c^	102.45^c^	0.59^d^
T16—*P*. *ultimum*	0.78^d^	0.59^ij^	0.70^ef^	39.71^c^	102.42^c^	0.60^d^
LSD (*P ≤ 0.05*)	0.039	0.029	0.027	1.807	3.996	0.031

Values are mean of three replications ± SD (standard deviation). Values in each column followed by same letter (s) are not statistically different by LSD (P ≤ 0.05).

* *F. oxysporum—F. oxysporum f. sp. Lycopersici*.

## Discussion

Members of pink-pigmented facultative methylotrophs occupies different habitats such as leaf surface, soil, water, and air. The plant-methylotroph association could be attributed to the unique ability of these bacteria to grow at the expense of methanol, a cell wall pectin degradative product from plants. PPFM has the ability to oxidize methanol using the methanol dehydrogenase enzyme. The mxaF gene encodes the large subunit of this enzyme, which is key in methylotrophic metabolism. In recent years, interaction study have shown that PPFM bacteria can increase plant protection against phytopathogen attack (Benhamou et al., 2000). In the current study, PPFM bacteria were isolated from various crop plants and used as a bio-control agent against tomato phytopathogens.

Bio-control ability of the PPFM isolates against phytopathogens were tested using three methods. One is by dual culture assay in which the PPFM isolate and pathogens were grown in same plate. In that the isolates PPO-1, PPT-1, and PPB-1, exhibited higher antagonistic potential against *F*. *oxysporum* f. sp. *lycopersici, S*. *rolfsii, P*. *ultimum*, and *R. solani*. The inhibitory effect of fungal growth observed in the current study in the dual culturing assay may be attributed to certain diffusible antifungal metabolites of the methylotrophic isolates (Montealegre et al., 2003). In the second method, the effect of PPFM volatile compound against phytopathogen was tested. The results revealed that PPFM bacterial isolates produced volatile antifungal compound which inhibited the growth of test fungal pathogens. In third method, both PPFM isolates and phytopathogens were simultaneously grown in liquid medium as co-cultures. The reduction in fungal biomass due to inhibitory effect of antagonistic PPFM was compared with fungal cultures grown in medium without antagonistic PPFM. Earlier a non-methylotrophic *Delftia lacustris* possessing nitrogen fixing trait capable of suppressing the growth of rice pathogens (*Xanthomonas oryzae* pv. *oryzae, R. solani*, and *Pyricularia oryzae*) was reported by Han et al. (2005).

The PPFM isolates capable of producing siderophopres proves their competitive advantage in the natural ecosystem by limiting the supply of iron and essential trace elements to the fugal pathogens as previously suggested by Indiragandhi et al. (2008). The inhibitory effect on the fungal pathogens tested may also be attributed to the salicylic acid production capability of PPFM isolates, as already evidenced in *Methylobacterium oryzae* CBMB20 inoculated *Pseudomonas syringae* pv. *tomato* challenged tomato plants (Indiragandhi et al., 2008). However, PPO-1, PPT-1, and PPB-1 methylotrophic bacterial isolates not producing HCN, eliminated the possibility of blocking the cytochrome oxidase system in fungal pathogen (Trivedi et al., 2008). Presence of chitinase and β-1,3-glucanase activities were previously reported in *Delftia* sp., *B. subtilis* and *B. cereus* (Chen et al., 2004; Jørgensen et al., 2009; Liang et al., 2014). Chitin and glucans those are responsible for the rigidity of fungal cell walls, thereby destroying cell wall integrity due to secretion of chitinase and β-1,3-glucanase limiting the growth of the pathogen (Chen et al., 2004; Jørgensen et al., 2009; Liang et al., 2014).

Methylotrophs oxidize methanol to formaldehyde via a key enzyme methanol dehydrogenase and the use of structural gene mxaF as a functional probe for methylotrophs is well-explained (Sy et al., 2001). Therefore, to evaluate the presence of methanol oxidation genes PCR amplification was performed with non-degenerate primers corresponding to highly conserved regions of mxaF gene sequences. Presence of mxaF gene and utilization of methanol clearly indicated the methylotrophic nature of isolates PPO-1, PPT-1, and PPB-1.

In the current study, none of the tested methylotrophic isolates exhibited hemolytic activity. This test was applied to investigate the potential pathogenicities of clinical and environmental isolates for plants and humans (Bevivino et al., 2002). In a previous study, Shin et al. (2012) observed the hemolytic behavior and resistance to antibiotics such as cefepime, amikacin, and gentamycin in opportunistic pathogenic *D. lacustris*. In the present study *D*. *lacustris* PPO-1 did not exhibit hemolytic activity and susceptibility toward various antibiotics. Shin et al. (2012) isolated *D*. *lacustris* from blood and bile fluids of human whereas *D*. *lacustris* PPO-1 was isolated from phylloplane of onion. Clinical *Burkholderia cepacia* isolates generally exhibited resistance to larger numbers of antibiotics and a higher degree of resistance to the single antibiotics than the environmental isolates (Bevivino et al., 2002). Clinical strains of *B*. *cepacia* secrete cytotoxic factors that allow macrophage and mast cell death in the presence of external ATP which appears to be lower in the environmental strain (Melnikov et al., 2000).

Hence, it is concluded that *D*. *lacustris* PPO-1 is probably not a pathogen. However, suitable molecular finger printing techniques should be devised in future to differentiate pathogenic clinical *D*. *lacustris* from environmental isolates. Chun and Bae (2000) demonstrated the use of *gyrA* sequences (coding for DNA gyrase subunit A) for accurate classification of *B*. *subtilis* and related taxa. In this study, isolate PPT-1 showed the amplified product size of 1000 bp which corresponds to the g*yrA* gene and confirmed as *Bacillus subtilis*. Though facultative methylotrophy has been documented in diverse heterotrophic genera (Hung et al., 2011), to the best of our knowledge, this is the first study to report the methylotrophy in *Delftia lacustris*. Nevertheless, the occurrence of pink pigmented *B*. *subtilis* was earlier reported, their methylotrophy was not documented (Oppong et al., 2000). However, the methylotrophy was reported only in non-pigmented *B*. *methylicus, B*. *methanolicus*, and *B*. *methylotrophicus* (Arfman et al., 1992; Madhaiyan et al., 2010). Hence, our study also first reports the methylotrophic pink pigmented *B. subtilis* PPT-1 and *B. cereus* PPB-1.

In the current study, the reduction of root rot and wilt disease incidences in the *D. lacustris* PPO-1, *B*. *subtilis* PPT-1, and *B*. *cereus* PPB-1 inoculated pathogen challenged tomato plants may be attributed to their direct antagonistic effect producing diffusible and volatile antibiotics as well as indirect induction of β-1, 3-glucanase, chitinase, PO, PPO, PAL, and catalase. ISR in several crops has been correlated with a two fold increase in activity of pathogenesis related PO and chitinase proteins in PGPR inoculated plants when challenged with pathogens (Nielsen et al., 1998; Xue et al., 1998; Nandakumar et al., 2001). Previously, several studies reported the induction of ISR like chitinase, PAL, β-1,3-glucanase, peroxidase, and PPO in rice, peanut, and tomato plants due to methylobacterial inoculation against various pathogens (Madhaiyan et al., 2004, 2006; Indiragandhi et al., 2008). Similarly, Choudhary and Johri (2008) explicated the mechanisms of *Bacillus* species as inducers of systemic resistance and Kloepper et al. (2004) reported induction of systemic resistance due to inoculation of *Bacillus* spp. in several crops including tomato. These defense proteins have the potential to hydrolyze the major components of fungal cell walls *viz*, chitin and β-1,3-glucans (Ren and West, 1992). The ubiquitous plant colonizing methylobacteria shown to be highly resistant to various abiotic stresses (Romanovskaya et al., 1996) could have enabled them for their effective epiphytic colonization in plants compared to other biocontrol agents.

## Conclusions

The finding of the present study not only supports the use of these pigmented facultative methylotrophic isolates as a potential biocontrol agents against tomato root pathogens but also expands our current knowledge on the taxonomy of facultative methylotrophic bacteria. Further field level performance testing of these methylotrophic isolates will confirm their biocontrol efficacy against root pathogens of tomato.

## Author contributions

VJ and VK—Isolated bacterial culture and conducted pot culture experiment; RA—conceived and designed the experiments; VJ and RA—analyzed the data; SK—Sequenced the bacterial culture; SUSU, TS, RK, KK, and SS—Manuscript preparation and editing.

### Conflict of interest statement

The authors declare that the research was conducted in the absence of any commercial or financial relationships that could be construed as a potential conflict of interest.

## Supplementary material

Figure S1 **Treatment details used in this experiments**. Filled arrow indicates that the respective column contains the bacterial treatment. Dotted arrows indicates the challenge inoculation of pathogen.

Figure S2 **Colony morphology of pink pigmented facultative methylotrophic isolates in Ammonium mineral salts medium supplemented with 0.5% methanol incubated at 28 ± 2° C for 5 days. (A)** *Bacillus subtilis* PPT-1; **(B)** *Bacillus cereus* PPB-1; **(C)** *Delftia lacustris* PPO-1.

Figure S3 **Amplification of ***gyr A*** gene**. L1, Negative control; L2, Methylotrophic isolate PPB-1 L3, Methylotrophic isolate PPT-1; L4, Positive control (*Bacillus subtilis* MTCC 121); M, Marker (0.5–10 kb DNA Ladder).

Figure S4 **Influences on methylotrophs on plant growth and diseases control in pot culture experiment**. T1—PPT-1 + *F. oxysporum* f. sp. *lycopersici*, T4—PPT-1 + *S. rolfsii*, T7—PPT-1 + *R. solani*, T12—PPO-1 + *P. ultimum*, T13—*F. oxysporum* f. sp. *lycopersici* T14—*S. rolfsii*, T15—*R. solani*, T16—*P. ultimum*.

Table S1 **Screening for antagonistic facultative methylotrophs against plant pathogens**.
