# Supplementary figures and images for: Control of Wilt and Rot Pathogens of Tomato by Antagonistic Pink Pigmented Facultative Methylotrophic Delftia lacustris and Bacillus spp
Source: Front Plant Sci. 2016 Nov 7;7:1626. doi: 10.3389/fpls.2016.01626 (PMC5097904; doi:10.3389/fpls.2016.01626)

Supporting Figure S1

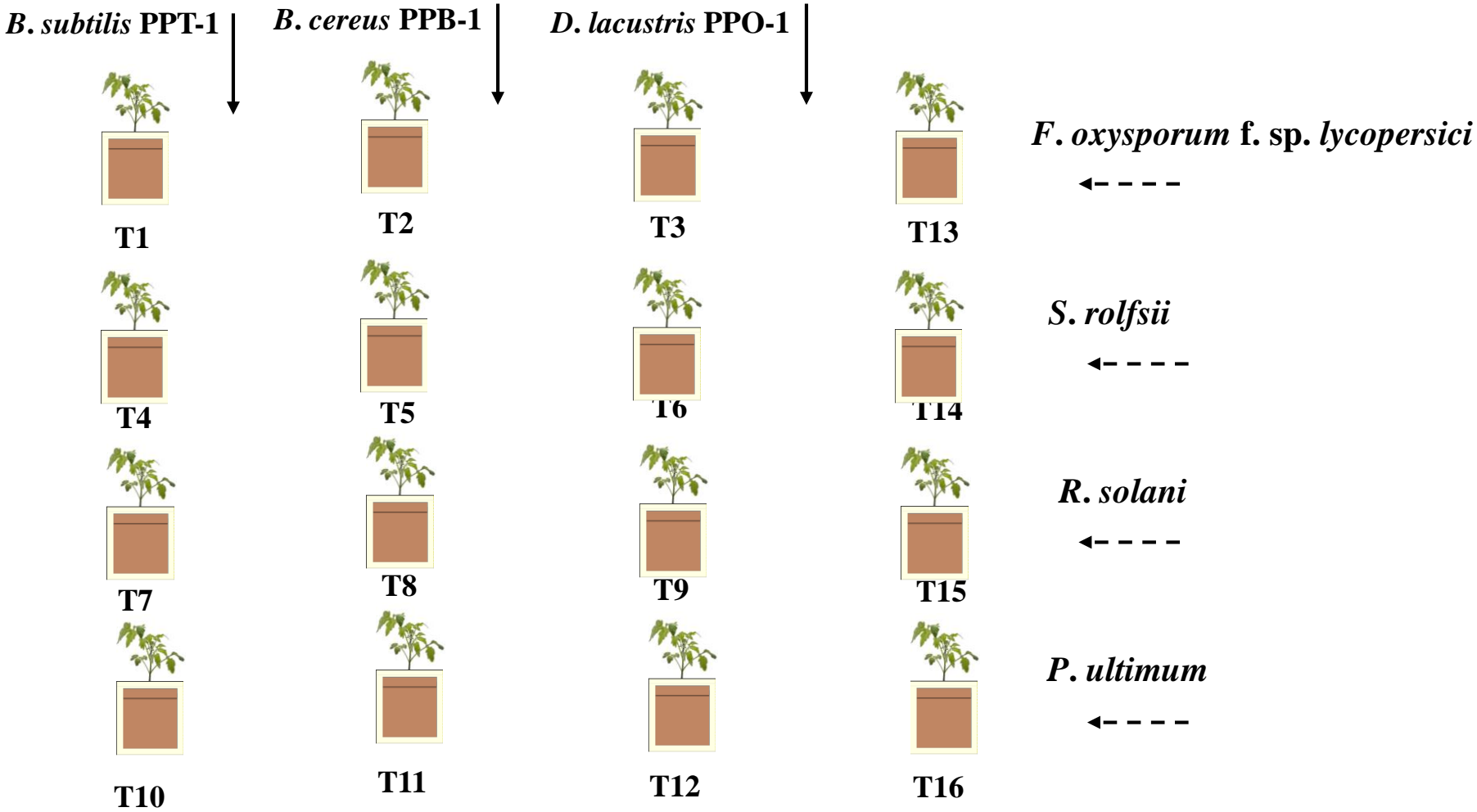

Supplement: Figure S1 — Treatment details used in this experiments. Filled arrow indicates that the respective column contains the bacterial treatment. Dotted arrows indicates the challenge inoculation of pathogen. [file Image1.PDF]

Supporting Figure S2

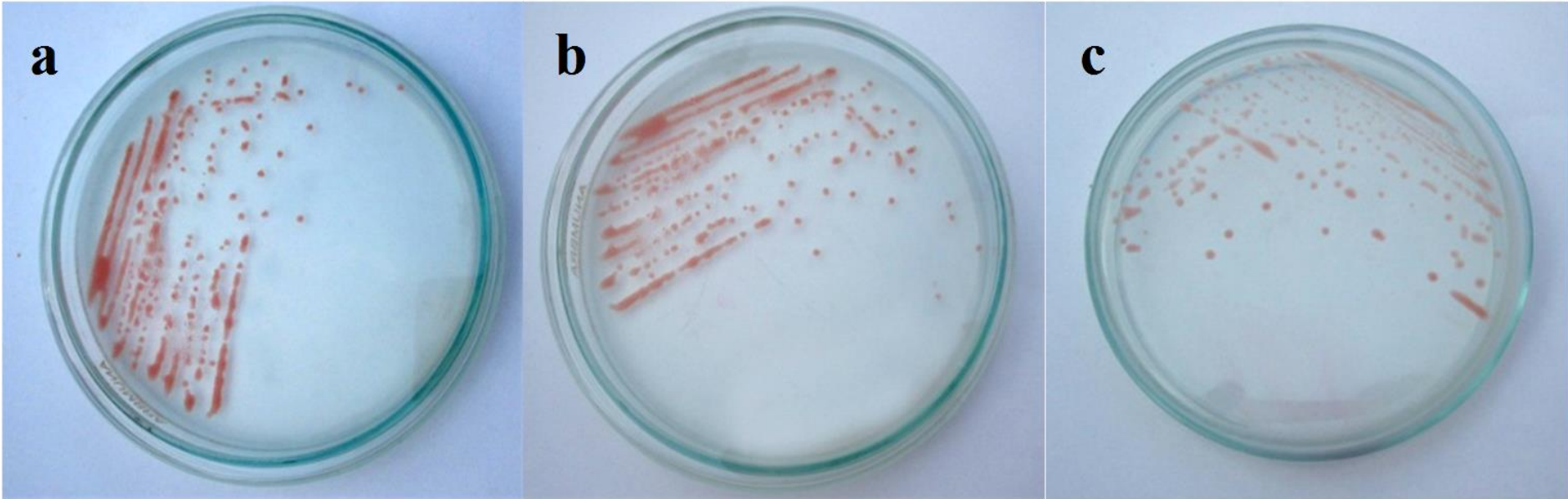

Supplement: Figure S2 — Colony morphology of pink pigmented facultative methylotrophic isolates in Ammonium mineral salts medium supplemented with 0.5% methanol incubated at 28 ± 2° C for 5 days. (A) Bacillus subtilis PPT-1; (B) Bacillus cereus PPB-1; (C) Delftia lacustris PPO-1. [file Image2.PDF]

**Supporting Figure S3**

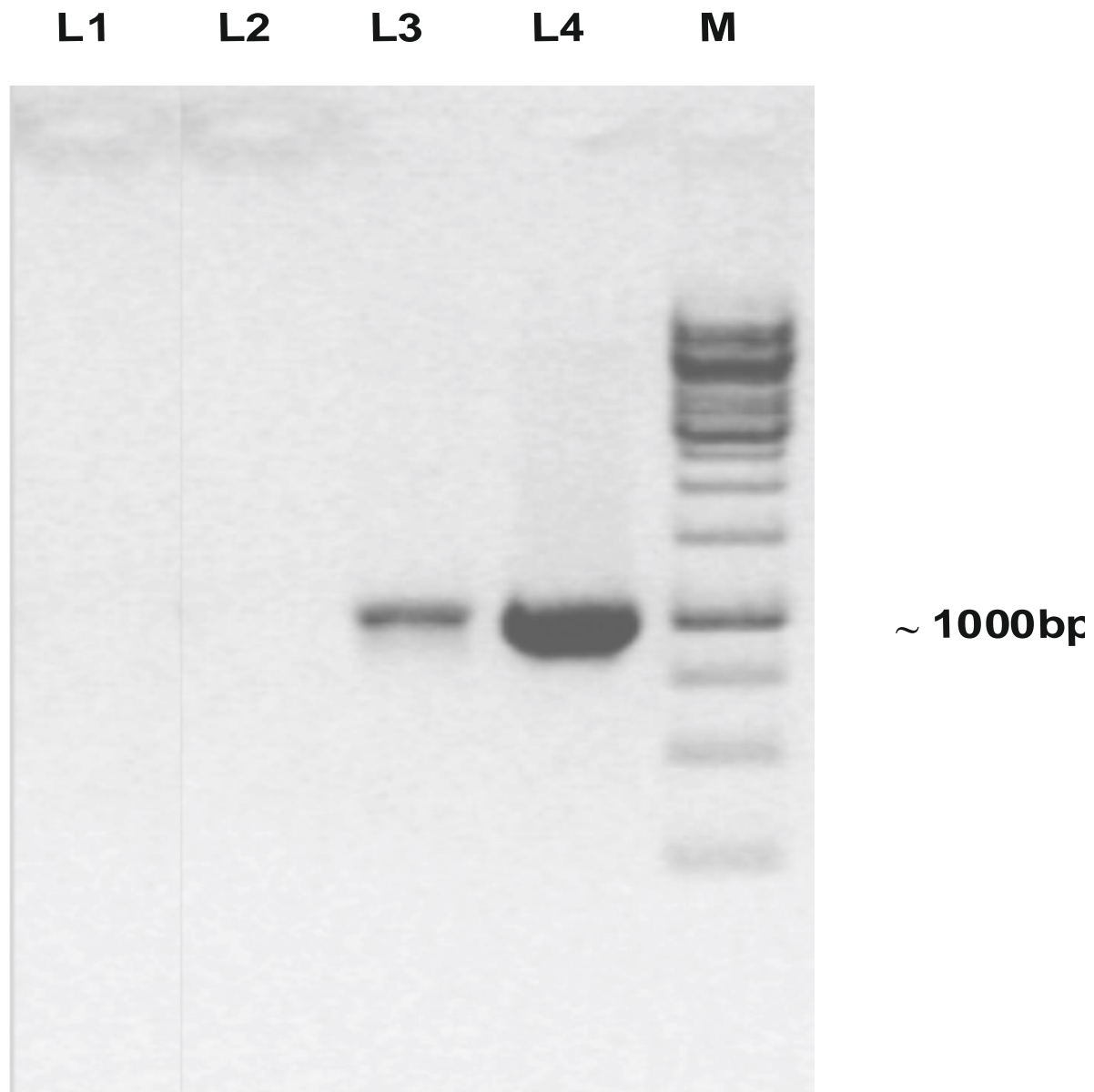

Supplement: Figure S3 — Amplification of gyr A gene. L1, Negative control; L2, Methylotrophic isolate PPB-1 L3, Methylotrophic isolate PPT-1; L4, Positive control (Bacillus subtilis MTCC 121); M, Marker (0.5–10 kb DNA Ladder). [file Image3.PDF]

**Supporting Figure S4**

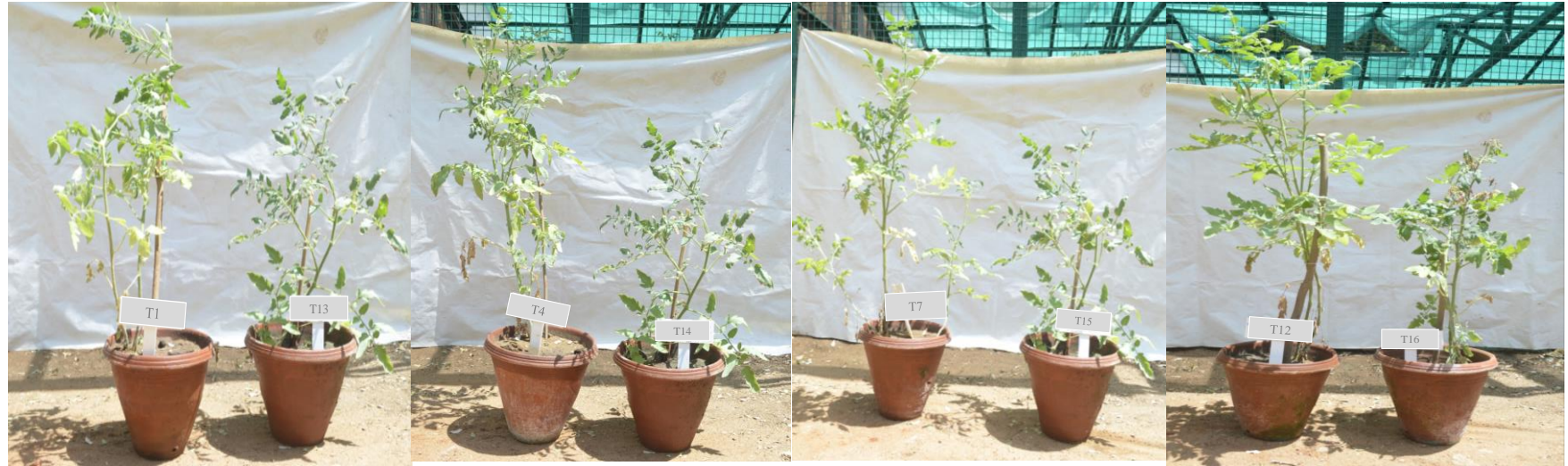

**T1      T13**

**T4      T14**

**T7      T15**

**T12      T16**

Supplement: Figure S4 — Influences on methylotrophs on plant growth and diseases control in pot culture experiment. T1—PPT-1 + F. oxysporum f. sp. lycopersici, T4—PPT-1 + S. rolfsii, T7—PPT-1 + R. solani, T12—PPO-1 + P. ultimum, T13—F. oxysporum f. sp. lycopersici T14—S. rolfsii, T15—R. solani, T16—P. ultimum. [file Image4.pdf]
