# Supplementary material for: Control of Wilt and Rot Pathogens of Tomato by Antagonistic Pink Pigmented Facultative Methylotrophic Delftia lacustris and Bacillus spp
Source: Front Plant Sci. 2016 Nov 7;7:1626. doi: 10.3389/fpls.2016.01626 (PMC5097904; doi:10.3389/fpls.2016.01626)
Supplement: Table S1 — Screening for antagonistic facultative methylotrophs against plant pathogens. [file Table1.pdf]

Table S1. Screening for antagonistic facultative methylotrophs against plant pathogens

Isolation source (Leaf)	Isolates	Pathogens			
		Fusarium oxysporum	*Rhizoctonia solani*	*Colletotrichum circinans*	*Alternaria solani*
Tomato	PPT-1	+	+	+	+
Jasmine	PPJ-1	-	-	-	-
Bhendi	PPB-1	+	+	+	+
Chilli	PPC-1	-	-	-	-
Onion	PPO-1	+	+	+	+
Ground nut	PPGR-1	+	+	+	+
Rose	PPR-1	-	-	-	-
Neem	PPN-1	+	+	+	+
Tamarind	PPTA-1	-	-	-	-
Prosopios	PPP-1	+	+	+	+
Brinjal	PPBJ-1	+	+	+	+
Marigold	PPM-1	-	-	-	-
Grasss	PPGS-1	+	+	+	+
Pomegranate	PPPO-1	-	-	-	-
Bouganivillea	PPBO-1	+	+	+	+
Sapota	PPS-1	-	-	-	-
Guava	PPG-1	+	+	+	+
Parthenium	PPPA-1	+	+	+	+
Papaya	PPPAP-1	-	-	-	-
Lime	PPL-1	+	+	+	+
