# Supplementary material for: Control of Wilt and Rot Pathogens of Tomato by Antagonistic Pink Pigmented Facultative Methylotrophic Delftia lacustris and Bacillus spp
Source: Front Plant Sci. 2016 Nov 7;7:1626. doi: 10.3389/fpls.2016.01626 (PMC5097904; doi:10.3389/fpls.2016.01626)
Supplement: Supplementary file 6 [file DataSheet1.PDF]

Supporting Information

Materials and Methods

I. Other antimicrobial traits assay

Siderophore production by PPFM bacterial isolates was tested on universal chrome azurol s (CAS) agar plates (Schwyn and Neilands, 1987). Briefly, 60.5 mg of CAS was dissolved in 50 mL of deionised water, and mixed with 10 mL of a Fe^{3+} solution (1 mmol L^{-1} $FeCl_3.6H_2O$, 10 mmol L^{-1} HCl). While stirring, this solution was slowly mixed with 72.9 mg of hexadecyltrimethylammonium bromide (HDTMA) previously dissolved in 40 mL water. The resulting dark-blue solution was autoclaved, cooled to 50°C and mixed with 900 mL sterile MM9 containing 15 g L^{-1} agar (kept at 50°C). This medium was allowed to solidify on Petri dishes, was inoculated with bacterial strains and incubated in the dark (28°C for 5 days). Formation of clear halo zones around colonies were considered as positive for siderophore production. Further, the siderophores produced by the test isolates was quantified by the method described by Reeves et al. (1983). Briefly, one ml of culture supernatant was mixed with 1 ml of HCl, 1 ml of nitrite molybdate and 1ml of NaOH followed by distilled water to make up the volume to 5 ml. Final absorbance was measured at 500 nm using 2,3 dihydroxybenzoic acid as the standard.

Qualitative assay for hydrocyanic acid (HCN) production was tested on King's B medium (Kremer and Souissi, 2001). Quantification of salicylic acid production in the culture supernatant was performed according to Meyer and Hofte 1997. Briefly, bacterial cultures were grown in casamino acids broth for 24 h at 100 rpm and 30°C in the dark. Subsequently, 100 µl of this culture was transferred to 25 ml of casamino acids broth and incubated for 36 h under the

same conditions. The culture broth was extracted with ethyl acetate and concentrated (1:3) under vacuum. Salicylic acid concentration was determined by adding 5 µl of 2 M $FeCl_3$ and 3 ml of water to 1 ml of concentrated extract. The absorbance was measured at 527 nm and compared with a standard curve of salicylic acid dissolved in ethyl acetate.

The enzyme β-1,3-glucanase activity was measured for bacteria grown in peptone medium containing 0.2% laminarin (from *Laminarindigitata*) for 4 d at 30 °C as per the method of Lim et al. (1991). The enzyme glucanase activity was determined as µg of glucose released min^{-1} mg of $protein^{-1}$. The chitinolytic activity of the methylobacterial isolates was determined as described by Indiragandhi et al. (2007). Briefly, the analysis was carried out by preparing an assay mixture consisting of 0.1 ml of the enzyme solution, 0.1 ml of 0.3% colloidal chitin, and 0.2 ml of a 0.1 mol McIlvaine buffer (pH 6.0). The reaction mixture was incubated at 35°C for 25 min and the reaction terminated by placing in a water bath for 15 min and the addition of 2.0 ml of a 1.5-mmol potassium ferricyanide reagent. The absorbance of the clear solution was measured at 420 nm (UV-spectrophotometer, Shimadzu, Japan). The activity was calculated using a standard curve generated from known concentrations of N-acetyl glucosamine (NAG). One unit of chitinase activity was defined as the amount of enzyme that liberated 1µmol of NAG released h^{-1} mg of $protein^{-1}$.

II. gyr A gene

Bacterial gyrA gene is a potential chromosomal marker for the phylogenetic identification of *Bacillus* genera. The genomic DNA from *B. subtilis* PPT-1 and *B. cereus* PPB-1was extracted using the method as described previously (Sambrook et al., 1989). A *gyrA* gene from isolates PPT-1 and PPB-1 were PCR amplified by using the primers *gyrA*-f (5'-CAGTCAGGAAA'IGCGTACGTCCTT-3') *gyrA*-r (5'-CAAGGTAATGCTCCAGGCATTW-3').

The amplified products were analyzed by electrophoresis in 1.5 % agarose gel. After separation of the PCR products in agarose gel, presence of *gyr A* gene was viewed and photographed using Alpha Imager TM1200 gel documentation and analysis system.

III.Detection of methanol dehydrogenase gene (mxaF)

The total genomic DNA from *D. lacustris* PPO-1, *B. subtilis* PPT-1 *B. cereus* PPB-1 and *Methylobacteriumextorquens* AM-1 (kindly provided by C. G. Friedrich University of Dortmund, Germany) was isolated by using the method as described previously (Sambrooket al., 1989). Methanol dehydrogenase (mxaF) gene was amplified from DNA samples in 50µl reaction mixture containing 20 ng genomic DNA, 200 mMdeoxynucleotide triphosphates (dNTPs), 100pmol primers (forward primer mxa f1003 5'-GCG GCA CCA ACT GGG GCT GGT-3' and reverse primer mxa r1561 5'-GGG CAG CAT GAA GGG CTC CC-3'), 1.5 mM $MgCl_2$, 1.25 U Taq DNA polymerase (Himedia, India), and 1X buffer. The polymerase chain reaction (PCR) amplification was performed in an Eppendorf gradient thermal cycler after an initial denaturation step at 95 °C for 5 minutes followed by 30 cycles (92 °C for 1 min, 55 °C for 1 min, and 72 °C for 1 min, and a final extension step at 72 °C for 5 min).

Reference

Indiragandhi, P., Anandham, R., Madhaiyan, M., Poonguzhali, S., Saravanan, V.S., Kim, G.H., et al. (2007). Cultivable bacteria associated with larval guts of prothiofos resistant susceptible and field population of diamond back moth, *Plutella xylostella* and their potential for host nutrition and antagonism. *J. Appl. Microbiol.* 103, 2664-2675.

Kremer, R.J. and Souissi, T. (2001). Cyanide production by rhizobacteria and potential for suppression of weed seedling growth. *Curr. Microbiol.* 43, 182–186.

Lim, H., Kim, Y., and Kim, S. (1991). *Pseudomonasstutzeri* YLP-1 genetic transformation and antifungal mechanism against *Fusarium solani*, an agent of plant. *Appl. Environ. Microbiol.* 57, 510 – 516.

Meyer, G.D., and Hofte, M. (1997). Salicylic Acid Produced by the Rhizobacterium *Pseudomonas aeruginosa* 7NSK2 Induces Resistance to Leaf Infection by Botrytis cinerea on Bean. *Phytopathology* 87, 588-593.

Reeves, M.W., Pine, L., Neilands, J.B., and Balows, A. (1983). Absence of siderophore activity in *Legionella* species grown in iron-deficient media. *J. Bacteriol.* 154, 324–327.

Sambrook, J., Fritsch, E.F., and Maniatis, T. (1989). Molecular cloning: A laboratory manual *In* Ford, N., Nolan, C., Ferguson, M., Ockler. M., (eds.), Cold spring harbor laboratory, Press, New York, USA.

Schwyn, B. and Neilands, J.B. (1987). Universal chemical assay for the detection and determination of siderophores. *Anal. Biochem*. 160, 47–56.
